# Implying implausibility and undermining versus accepting peoples’ experiences of suicidal ideation and self-harm in Emergency Department psychosocial assessments

**DOI:** 10.3389/fpsyt.2023.1197512

**Published:** 2023-08-30

**Authors:** Clara Bergen, Lisa Bortolotti, Rachel Kimberley Temple, Catherine Fadashe, Carmen Lee, Michele Lim, Rose McCabe

**Affiliations:** ^1^School of Health and Psychological Sciences, City, University of London, London, United Kingdom; ^2^Department of Philosophy, University of Birmingham, Birmingham, United Kingdom; ^3^McPin Foundation, London, United Kingdom; ^4^Department of Psychology, University of Exeter, Exeter, United Kingdom; ^5^Department of Psychology, University College London, London, United Kingdom

**Keywords:** suicide, clinical communication, risk assessment, mental health, crisis care, Emergency Department (ED), conversation analysis (CA)

## Abstract

**Background:**

Patients seeking emergency care for self-harm and suicidality report varying experiences from being believed and taken seriously to not being believed and taken seriously. Epistemic injustice provides a conceptual framework to explore how peoples’ experiences of self-harm and suicidality are believed or not. We use an empirical method –conversation analysis – to analyze *epistemics* in clinical communication, focusing on how knowledge is claimed, contested and negotiated. In courtroom, police and political interaction, conversation analysis has identified communication practices implying implausibility in a person’s story to contest and recharacterize their accounts.

**Aims:**

To investigate communication practices in Emergency Department (ED) biopsychosocial assessments that may (1) undermine, imply implausibility and recharacterize or (2) accept peoples’ experiences of suicidal ideation and self-harm.

**Methods:**

Using conversation analysis, we micro-analyzed verbal and non-verbal communication in five video-recorded biopsychosocial assessments with people presenting to the ED with self-harm or suicidal ideation, and conducted supplementary analysis of participants’ medical records and post-visit interviews. We present three cases where experiences were not accepted and undermined/recharacterized and two cases where experiences were accepted and validated.

**Results:**

When peoples’ experiences of suicidality and self-harm were not accepted or were undermined, questioners: did not acknowledge or accept the person’s account; asked questions that implied inconsistency or implausibility (“Didn’t you tell your GP that you were coping okay?”); juxtaposed contrasting information to undermine the person’s account (“You said you were coping okay before, and now you’re saying you feel suicidal”); asked questions asserting that, e.g., asking for help implied they were not intending to end their life (“So when you called 111 what were you expecting them to do”); and resistinged or directly questioned the person’s account. Multiple practices across the assessment built on each other to assert *that the person was not suicidal, did not look or act like they were suicidal; that the person’s decision to attend the ED was not justified; that an overdose was impulsive and not intended to end life; asking why the person didn’t take a more harmful medication to overdose; that self-harming behaviors were not that serious and should be in the person’s control*. Alternative characterizations were used to justify decisions not to provide further support or referrals to specialist services. At times, these practices were also delivered when speaking over the patient. When peoples’ experiences were accepted, practitioners acknowledged, accepted, validated suicidality/self-harm and introduced a shared understanding of experiences that patients found helpful. Non-verbal feedback such as nodding and eye contact was central in acceptance of patients’ accounts.

**Conclusion:**

These findings advance our understanding of *how* peoples’ experiences of suicidality or self-harm are undermined or accepted in mental health encounters in the ED. They have important clinical implications: patients report that when their experiences are not accepted or undermined, this makes them more distressed, less hopeful about the future and discourages future help-seeking when in crisis. Conversely, acknowledging, accepting and validating suicidality/self-harm and introducing a new ways of understanding peoples’ experiences may make people less suicidal and more hopeful, generates shared understanding and encourages future help-seeking.

## Introduction

Self-harm and suicide are public health priorities worldwide. In the UK, 1 in 5 adults has experienced suicidal thoughts (1) and 1 in 16 has self-harmed (2). Patients seeking emergency care for self-harm and suicidality report varying experiences from being believed and taken seriously to not being believed, not being taken seriously and feeling judged for seeking help (3). This is consistent with experiences of people seeking wider mental health support, i.e., they are sometimes not believed and their experiences are not taken seriously by healthcare practitioners (4–7). Disclosures of suicidality and self-harm may also be taken less seriously for certain groups of people, such as women and older adults nearing the end of life (8, 9). Interactions with healthcare practitioners can shape peoples’ perceptions of whether they need and deserve medical attention (10). People describe a fear of being seen as “faking” or “just wanting attention” as a major barrier to seeking mental health care (11).

The fields of Philosophy and Sociology have theoretical and empirical tools for unpacking whether peoples’ experiences are accepted or downplayed, dismissed and disbelieved. In the field of Philosophy, there has been increasing interest in the notion of *epistemic injustice*, which includes testimonial and hermeneutical injustice (12). According to the notion of *testimonial* injustice, a person’s reports are dismissed or challenged because a feature of the person’s identity triggers a negative stereotype, which leads to denying credibility and authority to that person as a knower. In other words, the person is thought to be unreliable in producing or sharing knowledge and thus the person’s reports are overlooked, even when these are reports of the person’s own experience. Examples would be discounting a woman’s suggestions on how to conduct an experiment in a lab due to the stereotype that women are not good at science; or discounting a teenage patient’s report that they feel suicidal due to the stereotype that teenagers are overly dramatic.

Another aspect of epistemic injustice is hermeneutical injustice. This is where a person is denied the conceptual resources to understand their own experience. An example would be how women who live in a misogynistic society in which the concepts of sexual harassment or domestic abuse are not available, lack the opportunity to understand their own adverse experiences as experiences of harassment and abuse.

Although the original notion of epistemic injustice has been developed to explain power asymmetries in social interactions due primarily to sexism and racism, the concept has recently been applied to the mental health context, where negative stereotypes can be associated with people seeking mental health treatment or with those diagnosed with mental illness (13). For instance, when reporting their own experiences, people may not be taken seriously due to having a history of psychotic symptoms (14) and are not credited with the capacity to understand and share their experiences. Historically, within traditional psychiatric diagnostic frameworks, psychotic experiences have been considered not real. However, more recent approaches reframe psychotic experience as “real” to the person even if not experienced by others.

From a philosophical perspective, applying the concept of epistemic injustice to the clinical encounter enables us to conceptualize the attitude of an epistemically privileged party – not as a lack of respect or a failure of empathy (which would not be specific enough) – but as an act of injustice toward the party who is epistemically subordinate. The injustice amounts to assigning reduced credibility to a patient’s reports, effectively preventing the perspective of the patient from contributing to shared knowledge and decision making. As epistemic injustice concerns knowledge first and foremost, this does not simply tell us that dismissing a person’s perspective due to prejudice is morally objectionable. Rather, it is problematic from an epistemic point of view because the opportunity to gather knowledge that would benefit both parties and society at large is missed.

When one party has expertise that the other party lacks, epistemic injustice does not rule out the possibility of disagreement between the parties. Rather, it situates disagreement in a context where both parties are recognized as agents with a valuable perspective. A practitioner will have clinical experience and expertise that can be harnessed to identify the best means of support for the person. A patient may lack clinical expertise, but has insights deriving from their experience of living with a mental health problem, including, e.g., how they reacted in the past to treatment options. In a particular domain, one party may enjoy greater authority, but both perspectives are valuable and worthy of attention.

Epistemic injustice as such is not an on-off concept, but the extent to which a person’s perspective can be taken into account – and valued – admits of degrees and the framework allows for this. Epistemic injustice is based on the fact that the subordinate party is an epistemic agent and agency can be manifested in more or less sophisticated ways: some aspects of agency may not be fully developed, e.g., in a child or may be compromised by poor mental health. As such, epistemic injustice provides a conceptual framework to explore how peoples’ experiences of self-harm and suicidality are discussed in mental healthcare clinical encounters. This conceptual framework can be paired with an empirical method developed in sociology – conversation analysis – to analyze *epistemics* in interaction. This involves analyzing how knowledge is claimed, contested and negotiated in communication (15, 16). Conversation Analysis has been used to micro-analyze how knowledge is negotiated in a range of naturally occurring video-recorded social interactions [e.g., Heritage (15–17) and Stivers et al. (18)].

In interpersonal communication, speakers continually mark levels of knowledge about a topic relative to one another (16). For example, asking a question (“How are you feeling?”) can mark a lower level of knowledge on the topic (how they feel), relative to the person being asked. Similarly, asserting information (“I’ve been feeling really down.”) can mark greater knowledge relative to the person being spoken to. Relative knowledge is not static: it shifts constantly during interaction depending on the topic being discussed (15, 16). For example, a healthcare practitioner might indicate they have more knowledge relative to the patient about what medication is appropriate to prescribe.

**FIGURE 1:**
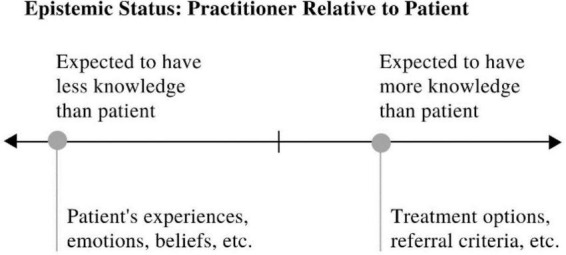
Linear representation of epistemic status with illustrative examples.

Sociologists distinguish between epistemic *status* and epistemic *stance* (16). Epistemic status involves *expectations* of knowledge, based on roles, e.g., doctor/patient, teacher/student, and experiences such as having studied a topic or having witnessed an event (Figure 1). For example, a teacher would typically be expected to know more about the topic of a lesson relative to a student. Similarly, a doctor would be expected to know more about diagnosis than a patient. This would mean that the teacher/doctor had a higher *epistemic status* than the student/patient on that topic. While a doctor would have higher epistemic status than a patient with respect to diagnosis, a patient would have higher epistemic status than a doctor on their experiences and emotions.

**FIGURE 2:**
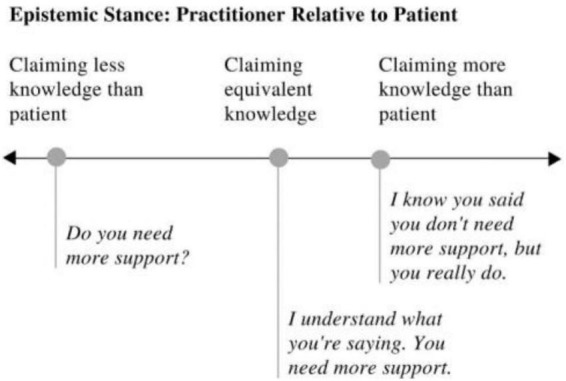
Linear representation of epistemic stance with illustrative examples.

In contrast, epistemic stance involves *communication* of knowledge (Figure 2). For example, when a teacher corrects a student, they take a higher epistemic stance, or implicitly communicate that they know more about that topic relative to the student. Similarly, when a doctor *informs* a patient of their diagnosis, they take a higher epistemic *stance* on the topic of that diagnosis.

In social interaction outside of institutional contexts, conversation analytic studies have demonstrated empirically that speakers and listeners orient toward speakers have primary rights to know and report on their subjective experiences (17). In healthcare interactions, patients typically have primary epistemic rights to know and report on their experience of symptoms while healthcare practitioners hold primary epistemic rights over diagnosis and recommending treatment options [e.g., Heritage and Robinson (19)]. Communication practices can be used to undermine peoples’ accounts of their experience. For example, there is a large body of literature examining communication practices in courtroom and police settings that seek and use evidence to undermine peoples’ accounts [see Drew (20), Antaki et al. (21), Stokoe et al. (22), and Jol and van der Houwen (23)]. For example, police questions may subtly imply inconsistency or implausibility, e.g., “Didn’t you just say that you were at home that evening?” (20, 22) or indicate objection or disagreement, e.g., “But how could you have known that?” (23). In political discourse and mass media, communication practices are used that contribute to a subtle erasure and rewriting of a person’s experience [see Clayman and Heritage (24)]. For example, politicians may repeatedly assert unsubstantiated information about other people or newscasters may assume or presuppose a different characterization of events in interviews (24).

There is little research on epistemic communication practices in mental health contexts. As there are typically no physical tests or investigations, mental health practitioners rely heavily on communication to assess mental state and ask patients about their mood, thoughts, feelings, behaviors and physical symptoms. Suicidal ideation involves thoughts and feelings of not wanting to live. Meanwhile, self-harm in the UK, is defined as intentional self-poisoning or injury, irrespective of motivation or the apparent purpose of the act (25). Self-harm can take many forms, including cutting, overdoses, burning, and hitting.

Emergency departments are often the first point of contact with healthcare services for people with suicidal ideation or self-harm who are at increased risk of suicide (26). Hence, they offer a key support system. Medical needs are treated by generalist emergency department practitioners and mental health practitioners from liaison psychiatry teams then offer a biopsychosocial assessment to assess the person’s current and future health and social care needs and make onward treatment referrals. This includes a suicide risk assessment in the context of a mental state examination to identify risk and protective factors to formulate suicide risk. Clinicians draw this together with information from other sources and make a structured professional judgment about the person’s level of risk (e.g., high, medium, and low), drawing on this to develop a management plan.

There is limited research on *how* assessments are conducted and on peoples’ experiences of risk assessment (27). Patients report varying experiences with some people reporting that they are believed while others report not being believed or that their experiences are not taken seriously (4, 5). Hence, the aim of this study was to use an empirical method, conversation analysis, to micro-analyze communication about suicidal ideation and self-harm in video-recorded biopsychosocial assessments in the Emergency Department (ED) to investigate communication practices used to (1) possibly undermine, imply implausibility and recharacterize peoples’ experiences of suicidal ideation and self-harm or (2) accept peoples’ experiences of suicidal ideation and self-harm.

## Materials and methods

The study involved detailed analysis of five video-recorded ED biopsychosocial assessments for self-harm and suicidal ideation, participating patients’ medical records and post-visit patient interviews. Self-harm was defined as intentional self-poisoning or injury, irrespective of motivation or the apparent purpose of the act (25).

### Ethics

The study was developed in collaboration with a lived experience advisory group and obtained ethical approval from London Central Research Ethics Committee (17/LO/1234).

### Treatment setting

The treatment setting was a liaison psychiatry team in the ED staffed by specialist mental health practitioners.

### Video data and participants

After presenting to the ED, participants were assessed by medical staff in the ED and had their medical needs treated before being referred for a biopsychosocial assessment with the ED Liaison Psychiatry team. The biopsychosocial assessment involved an assessment of needs and risks, including the risk of harm to self and determined whether the person would be admitted to hospital or was safe to be discharged along with support required from other community based services.

#### Consent

Before the biopsychosocial assessment, patients were approached by a liaison psychiatry practitioner who assessed capacity to give informed consent and asked if the person would be willing to speak to a researcher. There was a multi-step consent procedure due to people presenting in a mental health crisis. If patients agreed to be approached, a researcher explained the study and obtained written informed consent before the biopsychosocial assessment. The practitioner re-affirmed consent during the assessment, and the researcher re-affirmed consent 1–2 weeks after the assessment.

#### Data

Data were from three sources (1) a corpus of 46 video-recorded Liaison Psychiatry biopsychosocial assessments collected between September 2018 and April 2019 in an ED in England [see Xanthopoulou et al. (28) and Bergen and McCabe (29)]. Two GoPro cameras were placed in the assessment room and the assessment was recorded with no researcher present. (2) Each patient’s ED medical records including the written risk assessment and patient care notes were obtained after the assessment. (3) Patient participants were interviewed 2 weeks and 3 months after the assessment. A semi-structured interview explored patients’ experiences of the assessment and their health and treatment after the assessment.

Detailed notes were taken summarizing the content of all video-recorded assessments. These notes were reviewed to identify assessments in which practitioners did not accept the patient’s description of their experience of suicidal thoughts or self-harm and introduced an alternative characterization. Three assessments were selected as having particularly clear and recurring examples of this phenomenon. Two cases were then identified to compare these findings with communication when practitioners accepted peoples’ experiences. Ultimately, this article focuses on five assessments: three assessments in which the patient’s experiences were recharacterized by the practitioner and two assessments in which the patient’s experiences were accepted by the practitioner.

Patients presented with suicidal ideation (*N* = 3) or after a suicide attempt (*N* = 2). Patients identified as white British (*N* = 4) and Indian (*N* = 1), male (*N* = 2) and female (*N* = 3), and were aged between 18 and 55. Five Psychiatric Liaison Practitioners (PLPs) participated: two were mental health nurses, two were occupational therapists, and one was a social worker. PLPs identified as white British (*N* = 4) and African (*N* = 1), male (*N* = 2) and female (*N* = 3), and were aged between 40 and 60.

### Data analysis

#### Video recordings

Conversation analysis (30) was used to micro-analyze verbal and non-verbal communication. We sought to identify when a person’s experiences were not acknowledged or accepted and the specific communication practices used to subtly recharacterize a person’s description of their experience. We analyzed communication practices over the course of an assessment as individual practices may not immediately be seen as recharacterizing the person’s experiences but multiple practices over the course of an assessment could be hearable as seeking and using evidence to discredit a person’s characterization of their experience and introduce an alternative characterization.

We drew on conversation analytic findings from police, courtroom, and political settings to identify these practices. Data were also presented and discussed in data sessions to (1) a diverse group of five people with experience of receiving professional support for mental health and suicidal thoughts, and (2) a multidisciplinary group of six professionals from psychiatry, psychology, and philosophy.

We analyzed practitioner–patient communication about suicidal ideation and self-harm. We analyzed patient responses indicating lack of agreement with the practitioner’s utterances and questions including: explicit disagreement; correcting the practitioners’ talk and more subtle signs of patient disengagement including silence, minimal responses, quiet or flat voice quality, reduced eye contact, and not contributing to the forward progression of the assessment, i.e., not answering questions or sharing information to facilitate the practitioner conducting the assessment [see Peräkylä et al. (31)].

A range of communication practices were identified. The main practices are listed in Table 1 and are discussed in detail using data extracts below.

**TABLE 1:** Communication practices used to recharacterize patients’ experiences.

Communication practice	Studies in other settings	Examples (hypothetical, simplified)
**Not accepting or acknowledging** a person’s characterization of events	Marquez-Reiter et al. (32)	Pat: I’m feeling suicidal. Pra: **[writing notes, no response]**
Question **implies inconsistency or implausibility**	Stokoe et al. (22)	Pat: I’m feeling suicidal. Pra: **Didn’t you tell your General Practitioner** you were coping okay?
Question **embodies a compromising respons**e that could be used against the person’s characterization	Drew (20)	Pat: I’m feeling suicidal. Pra: But you’ve felt like this before and **you got through it, right?**
Statement **juxtaposes information** that may undermine characterization or strengthen argument for alternative characterization	Drew (20)	Pat: I’m feeling suicidal. Pra: **You said** you were coping okay before, and **now you’re saying** you feel suicidal.
**Asserting an alternative** characterization (sometimes repeatedly)	Clayman and Heritage (24)	Pat: I’m feeling suicidal. Pra: But overall **you’ve been coping okay**.
**Questioning or resisting** a person’s characterization of events	Waring (33)	Pat: I’m feeling suicidal. Pra: **Really?**

To supplement conversation analysis of the video-recordings, we also explored and triangulated data from other sources:

1. Medical records: assessment summaries written by practitioners in the medical records after the assessment. Risk assessments and notes entered after the assessment in the patient’s medical records were reviewed to identify how practitioners described the patient’s account of their suicidal thoughts/feelings and self-harm This data was analyzed on a simple descriptive level and we report direct quotes from these sources.
2. Patient interviews: 2 week and 3 month post-visit patient interviews were reviewed to integrate patients’ experiences on the assessment and interaction with the practitioner. Patient quotes are provided.

## Findings

We present five cases in-depth: three cases undermining peoples’ experiences and two cases accepting and validating peoples’ experiences.

## Implying implausibility and undermining peoples’ experiences

Practitioners used specific communication practices to recharacterize, downplay and undermine patients’ descriptions of their experiences. In each case, multiple communication practices built on one another to support an alternative characterization. In this section, we explore how this evidence is built across each biopsychosocial assessment and where patients’ primary epistemic rights to know and describe their subjective experience is undermined across three cases.

### Case 1 Patrick: recharacterizing the patient’s experiences of misery, feeling suicidal, and undermining a decision to seek help in the ED

Patrick was brought to the ED by his university counseling service after he disclosed thoughts of suicide. Here, we examine brief extracts from Patrick’s video-recorded biopsychosocial assessment and 3-month post-visit interview. At the start of the assessment, Patrick confirmed that he was “feeling suicidal” (transcript not shown) and described feeling fearful that he would end his life if he left his flat (see Extract 1). Transcription symbols are described in Appendix Table 1.

**Extract 1:** 

1	PR:	What would have happened if you
		had gone for a walk.
2	PT:	I don’t kno:w.=I think, (2.0)
		I hadn’t thought that far ahead,
3	PR:	Mm.=
4	PT:	=but li:ke (2.0) I was just-
		I figured, **if I leave here it’s**
5		**the e:nd. I’m gonna kill myself.**
		So.

Later, after Patrick describes his experience, the practitioner asks what happened when the university counseling service got involved (transcript not shown). Patrick’s answer is shown in Extract 2 (lines 51–53).

**Extract 2:** 

51	PT:	We had a conversation: and then (.)
		>they spoke about the<
52		possibility of going to hospital,=and
		I tho:ught, prob’ly a
53		good idea.
		…
61	PR:	So they spoke about that possibil-
		What (.) From your point-
62		What made them think that um
63		(1.0)
64	PR:	‘Cause they’re- they see you because
		of mental health
65		reaso:ns, (.) and **what made them think**
		**that their i:nput**
66		**wouldn’t be he:lpful for you.**
67		(0.5)
68	PR:	**an:d that it would be helpful for**
		>**you to come to hospital.**<
69		from your point of [view.
70	PT:	[They’re not- They’re not-
		I dunno.They’re
71		not trained in any of this kind of
		stuff. They’re kind of .hh
72		the go: between. Between (.) different
		places. And they
73		thought. (.) They’d be- I think- I
		think- >I mean I don’t
74		know< for certain because I didn’t
		ask them.
75	PR:	Mm.
76	PT:	But I think they tho:ught that (.)
		it would be good for me to
77		speak to someone (.) who knew what
		they were on about.
78	PR:	.hh I see. **So they felt that they**
		**didn’t have the- enough**
79		**trai:ning [to- to to talk to you and**
		**reassure you.**
80	PT:	[Yeah.
81	PT:	Mhm.

Patrick initially characterizes his decision to attend the ED as a “good idea” prompted by a recommendation from a university counselor (lines 51–53). The practitioner does not agree and instead asks a follow-up question (lines 61–69) indicating that it is not clear why it would be helpful for Patrick to come to hospital, and why his problems could not be addressed by the university counseling service. This introduces a potential alternative characterization, that attending the ED was not a good idea.

Patrick shows difficulty responding; after multiple restarts and expressions of uncertainty (lines 70–74, 76–66), he provides justification for the counselor’s recommendation. The practitioner summarizes the university counselor did not feel they had the training to “talk to you and reassure you” (lines 78–79). This implies that talking and reassurance would have been enough to address Patrick’s concerns, thereby positioning Patrick’s concerns as not warranting presentation to the ED.

When discussing the reasons underlying his suicidal thoughts, Patrick describes feeling miserable. In Extract 3 below, a second practitioner asserts that he is either not miserable at times or able to give the impression that he is enjoying things (lines 4–6) then implies that Patrick’s facial expressions provide evidence for this alternative interpretation (line 8) of Patrick’s feelings.

**Extract 3:** 

1	PT:	So I (.) feel like miserable kind of
		(.) sums it up,
2	PR:	**And yet in your fa:ce, you [know=**
3	PT:	[Yeah,
4	PR:	=**when you’re speaking. You’ve-**
		**You’ve got a variation. haven’t**
5		**you. of- of your expressio:n,=and you**
		**know you smi:le and**
6		**things like that.**
7	PT:	>Yeah,< ((no nonverbal response))
8	PR:	>**So you have times< when you clea:rly**
		**(0.3) aren’t miserable,**
9		**you’re sort of enjoying things, or**
		**you’re able to [give the**
10	PT:	[Mhm,
11	PR:	**impression [that you are enjoying**
		**thi:ngs,**
12	PT:	**[**Yeah, ((small nod))

The practitioner does not accept Patrick’s description of his emotions (feeling “miserable”) at line 2. Instead, she cites his facial expressions (“you smile” lines 4–6) as evidence of an alternative interpretation; he has “times when” he isn’t miserable (line 8) and is “enjoying things” (line 9). Presenting her observation of his demeanor (lines 4–6) as evidence that he is not always miserable, this challenges the patient’s description of his emotional state [see Stokoe et al. (22)].

The contrastive formulation (line 2) and assertion of the alternative interpretation that he is “enjoying things or able to give the impression that you are enjoying things” (lines 8–9), paired with a lack of acceptance at line 2 (e.g., *okay*), discount Patrick’s characterization. Patrick responds minimally (lines 3, 7, and 10), showing signs of disagreement and disengagement and passive participation, withdrawing from the conversation and not agreeing with the practitioner’s interpretation “when you clearly aren’t miserable, you’re sort of enjoying things” in line 10 until after the practitioner self-corrects “or you’re able to give the impression that you’re enjoying things” (lines 11–12).

In Extract 4, later in the same assessment, a different practitioner asks what plan Patrick would have had if he had not gone to the ED.

**Extract 4:** 

47	PR:	What- What plan would you have [had
		if you-
48	PT:	[I
		just- Well I’ve got a
49		few events on. ‘Cause I’m part of
		rugby skiing and tennis.
50		And they were all putting events on tonight I couldn’t go
51		to.
52	PR:	**I see. So could we safely say, you**
		**know. you wouldn’t end**
53		**your life?**
54		(1.0)
55	PR:	Or something that would have=
56	PT:	=What tonight?
57	PR:	Yeah. [Y-
58	PT:	[I wouldn’t have ended it
		toni:ght. ((shakes head))
59	PR:	((nods)) **You wouldn’t have. Okay. So**
		**maybe there was a bit**
60		**of miscommunication because they-**
		**they brought you he:re**
61		**because they were**
		**saying you were suicida:l, and=**
62	PT:	=No I ((nod)) am.=But [I-
63	PR:	** [You a:re.**
64	PT:	But I’ve- I feel I can (3.0) I mean I
		haven’t done it yet,
65	PR:	Mm. ((nods))

Patrick indicates he would have attended a sporting event, and the practitioner makes an inferential connection “So could we safely say…you wouldn’t end your life” (34) implying that his answer provides evidence that he would not have ended his life (lines 52–53). Patrick pushes back against the question by requesting clarification “What tonight?” (line 56), giving a repetitional answer (“I wouldn’t have”) (35), and qualifying that he would not have ended it *that night*.

The practitioner repeats Patrick’s statement without the qualification – sequentially deleting – “toni:ght” (“You wouldn’t have”) and makes another inferential connection (“So maybe there was a bit of miscommunication…”) (34). He asserts that it may have been a miscommunication when the university counseling center said Patrick was suicidal. Patrick immediately resists this, asserting “I am,” stating that he has not “done it yet” (lines 62 and 64).

Across the course of the assessment, the two practitioners undermine the legitimacy of Patrick’s decision to seek help (Extract 2) and recharacterize Patrick as “not always miserable” (Extract 3) and “not suicidal” (Extract 4). Ultimately, Patrick was advised to visit a self-help website and continue to access university counseling. Over the next 3 months, Patrick returned to the ED twice; once for suicidal ideation and once for a pharmaceutical overdose with suicidal intent. In his 3-month post-visit interview, Patrick reported that he would not have gone to the ED again, but was brought back by university counseling services.

### Case 2 Laura: recharacterizing the patient’s experience of suicidal intent to justify no referral

In Case 2, a practitioner recharacterizes Laura’s experience of suicidal ideation as brief and her act as impulsive. Table 2 summarises the communication practices used to characterize Laura’s suicide attempt as impulsive. This is then used to justify a decision not to refer the patient to mental health services (anonymized). In contrast to impulsive acts of self-harm, practitioners treated premeditated suicide attempts as relatively more serious.

**TABLE 2:** Communication practices recharacterizing Laura’s suicide attempt as impulsive.

Practitioner’s characterization of suicidal act: “an impulsive thing”	
**Practitioner communication practice**	**Examples from Extract 5**
Asking questions that anticipate a compromising response (20)	“And then I hear that you called the ambulance straight away?”
Asking questions that imply inconsistency or implausibility (22)	“So when you called 111 what were you expecting them to do:.”
Juxtaposing contrasting information (20)	“Thinking I wanna end my li:fe, … and then you called them. So they would get you the [help.”
Implying information provides evidence of an alternative characterization (21)	“A::h. So would you say you took the tablets, at the spur of the moment,” … “So it was a more of an impulsive thing, at the time,”

Laura was brought to the ED by ambulance after a pharmaceutical overdose. Earlier in the assessment, Laura said she visited her General Practitioner earlier in the day seeking mental health support but “they didn’t help me” (transcript not shown). She reported that she later took a pharmaceutical overdose because she felt “very suicidal.” She does not indicate that she took the overdose impulsively. In this section, we examine brief extracts from Laura’s video-recorded biopsychosocial assessment and documents in her medical file, including a summary letter written for Laura’s General Practitioner by the Liaison Practitioner.

**Extract 5:** 

1	PR:	And then >what was the< intention
		when you took the overdose.
2		What was=
3	PT:	=To kill myse:lf,
4	PR:	**To kill yourself. And then I hear that**
		**you called the**
5		**ambulance straight away? Or: 111**,
6	PT:	N::o, I got- I got on the phone with
		111 and then they got an
7		ambulance.
8	PR:	For you.
9	PT:	For- For- Yeah.
10		PR: **So when you called 111 what were**
		**you expecting them to do:.**
11	PT:	All I expect- All I expected them to
		get an ambulance out to
12		me to be honest? That’s [(the way it
		works)
13	PR:	**[A::h. So**
		**would you say you took the**
14		**tablets, at the spur of the moment,**
15	PT:	Well I [took the tablets and then
		later
16	PR:	**[Thinking I wanna end my li:fe,**
17	PT:	on, I told [them how many tablets I
		had,
18	PR:	**[And then-**
19	PR:	**And then you got worried that you**
		**wanted to die, and then you**
20		**called them.=**
21	PT:	=Yeah.
22	PR:	**So they would get you the [help. Is**
		**that**
23	PT:	[Yeah.
24	PR:	**how, [Is that how it worked,**
25	PT:	[Sort of, yeah.
26	PR:	Yeah okay.
27	PT:	I sort of wanted to di:e,
28	PR:	Yeah. ((nod))
29	PT:	Sort of didn’t. Because I have the
		two kids to live fo:r,
		… ((discuss family relationships))
51	PR:	**So it was a more of an impulsive**
		**thing, at the time,**
52	PT:	It was just I- I’d had enough. Of
		people like Kate picking
53		on me.

In response to the practitioner’s question in lines 1–2, Laura states her intention was “to kill my:self” (line 3). The practitioner does not accept Laura’s answer (line 4) and asks her to confirm that she called for an ambulance “straight away.” The question grammatically anticipates a compromising response, i.e., a response that would indicate she quickly sought life-saving support. When Laura does not immediately confirm (lines 6–7), the practitioner pursues, asking a question (“what were you expecting them to do:.” line 10) that directly implies inconsistency between “wanting to end your life” and “calling 111” for help (22).

The practitioner makes an inferential connection (“So would you say,” line 13) (34) between Laura’s answer and the characterization that she took the tablets “spur of the moment” (line 14). The practitioner does not invite Laura to describe her thought process. He instead invites Laura to confirm a characterization that her overdose was impulsive, which would be considered lower risk relative to a premeditated attempt. Laura does not agree [lines 15/17, see Schegloff and Lerner (36)] and asserts she disclosed the overdose “later on.” The practitioner speaks over Laura in overlap (lines 13, 14, 16, and 18) as he continues to describe his characterization of events (“and then you got worried…”) and does not acknowledge Laura’s talk (lines 16, 18–20, and 22) [see Jefferson (37), p. 319].

Laura agrees with aspects of the practitioner’s description (“you called them. = So they would get you the help” lines 21 and 23), but when the practitioner asks her to confirm the overall characterization (including taking the tablets “spur of the moment”), she indicates it is not completely accurate (“Sort of,” line 25). She again attempts to describe her experience with conflicting feelings of suicidality and emphasizes the factors contributing to her decision to ultimately call for an ambulance as she has “two kids to live for” (line 29). The recharacterizations offered by the practitioner (that Laura wanted to die momentarily, then changed her mind and contacted an ambulance) does not leave space for the possibility that Laura may have experienced conflicting thoughts of suicide, both wanting to die and not wanting to die simultaneously.

Laura never agrees with the characterization “spur of the moment.” The practitioner later asks Laura to confirm that the overdose was “an impulsive thing” (line 51). Laura again does not accept this characterization and describes reaching a point where she had “had enough” (line 53).

In the discharge letter to Laura’s General Practitioner, the Liaison Psychiatry Practitioner writes: *[Laura] told us that [she] took the overdose impulsively because [she was] “Fed up with people picking on [her], especially [Kate].”*

Extract 6 occurs a little later in the same biopsychosocial assessment. The practitioner is asking a series of questions assessing to what extent the overdose was pre-planned (see lines 1–2).

**Extract 6:** 

1	PR:	And the co-codamol. Was- Was it there
		for your pa:in,
2		or wh- why: was it in your house.
3	PT:	Uh well I originally had it for pain
		relief.=
4	PR:	=A[h.
5	PT:	[But then I (.) took a ((inaudible))
		of i:t, and I took an
6		overdose.
7	PR:	((nod)) I see. **Why didn’t you take**
		**your overdose on your:**
8		**Depakote [and- and other: (.)**
		**medications,**
9	PT:	[((shakes head))
10	PT:	Because I didn’t think it will:
		have effect.

Laura explains that she purchased the co-codamol for pain relief (line 3). The practitioner then asks Laura to justify why she did not overdose on her prescribed medications, naming one particularly harmful medication (lines 7–8). The question implies implausibility that it was really Laura’s intention to end her life ().

Extract 7 occurs later in the same assessment. In Extract 7, the practitioner characterizes Laura’s suicide attempt as “impulsive” as he resists her suggestion of accessing a rapid response team if in crisis.

**Extract 7:** 

8	PR:	And would you ask for help if
9		those thoughts came back and,
10	PT:	I might ring the response team in.
11		To make sure I’m not taking
12		overdoses [and-
13	PR:	[I- ((nods))
14	PT:	to make sure ((inaudible)) it’s
15		alright. [Yeah-
16	PR:	**[You want the rapid**
17		**response team.**
18	PT:	Yeah. If there- If there is any,
19		[I don’t- I don’t know.
20	PR:	**[Well we’ll talk about that but-**
21	PT:	There was one where I used to live,
22		[A rapid response team,
23	PR:	[Yeah. **I can appreciate that you**
24		**feel this but until Kate upset you,**
25		**you’ve been coping generally okay,**
26	PT:	Yeah.
27	PR:	**And then this happened and then**
28		**caused this impulsive um behavior.**
29		To kind of uh-
30	PT:	Yeah.
31	PR:	You took the overdose. So at this
32		point in time you say you don’t have
33		any plans to do anything to cause you
34		harm.
35	PT:	No.
36	PR:	((transitions to further risk
		assessment questions))

The practitioner asks whether Laura would ask for help if she had suicidal thoughts (lines 8–9). Laura responds that she might ring the rapid response team (lines 10–15). The practitioner asks Laura to confirm (lines 16–17), indicating this is problematic (38) and flags that this may not be facilitated.

The practitioner acknowledges that Laura wants support from the rapid response team (lines 23–24 re lines 16–17) and speaks over the patient in interjacent overlap (lines 20 and 34). He asserts that until the triggering event Laura was “coping generally okay” (lines 24–25). He frames her overdose as “impulsive… behavior” that was “caused” by Kate (lines 27–29). Laura minimally agrees (lines 26 and 30) and the practitioner requests re-confirmation that she has no plans to harm herself (lines 31–34), a leading question that is designed for Laura to confirm she does not have plans to harm herself (39–41). This all works to build a case that the Rapid Response Team is not needed [see Anonymized (42)].

After Laura states that she has no plans to harm herself in response to the leading question, the practitioner transitions back to suicide risk assessment. Later, the practitioner recommends speaking to a friend or calling a charity helpline if she finds herself in a similar situation. In the risk assessment document, the practitioner writes: “*We have*… *encouraged you that if you are feeling low or have a fall out with someone you care about to try to talk to someone who will be kind, such as your landlord, or ring Samaritans. If you feel suicidal and this isn’t enough we have advised you to ring 111.”* There is no reference to the rapid response team. There was no patient interview, which we have found was often the case when a person had a negative experience of the biopsychosocial assessment in the ED.

### Case 3 Sasha: recharacterizing the patient’s experience of food restriction shifts the burden of care

As shown in the extracts above, recharacterizations can be built up during an assessment and can be cited to justify decisions not to provide specialist care. In the following extracts, we demonstrate how these recharacterizations can be used to shift the burden of care off of the healthcare system and back onto the patient (43).

Sasha attended the ED seeking help for worsening symptoms of obsessive compulsive disorder (OCD) restricting her food intake and feeling unable to control her intrusive thoughts of suicide and the need to do things in blocks of eight. This included dietary restriction to 800 calories per day, which had resulted in the rapid loss of about 22 pounds and a Body Mass Index bordering underweight. Eating disorder behaviors are viewed by some as an extreme form of self-harm. In the ED biopsychosocial assessment, Sasha asked about specialist support for eating disorders multiple times. In this section of the article, we share brief extracts from Sasha’s video-recorded biopsychosocial assessment and her 3-month post-visit interview. In Extract 8, Sasha describes her experience of food restriction.

**Extract 8:** 

1	PT:	Because: **my obsessive behaviors have**
		**been getting worse and**
2		**worse as well.=They’ve now kind of**
		**spread into: (1.0) um (.)**
3		**areas of my life like eating:,**
4	PR:	Mm. ((nod))
5	PT:	Um (.) yeah Steve said that he was
		really concerned,(.) about
6		(.) the weight that I’ve lost so
		[rapidly: and I
7	PR:	[Mm. ((nod))
8	PT:	can feel my heart slowing do:wn:, and
		**I can feel the physical**
9		**symptoms from it.**
10	PR:	Mm:.

Sasha describes her food restriction as an obsessive behavior stemming from her OCD (lines 1–3), thereby framing the behavior as a symptom outside her control. She emphasizes the speed of her weight loss, others’ concern, and the physical impact on her body (lines 5–6 and 8–9). She positions the food restriction as a concerning symptom for which she is seeking help. She describes her experience of food restriction again in Extract 9A.

**Extract 9A:** 

1	PR:	And and in terms of you:r
		understanding. What’s your diagnosis
2		Sasha,
3	PT:	Um: OCD, and (.) anxiety, I think,
		((shakes head))
4	PR:	Okay. ((nods))
5	PT:	((nods))
6	PR:	And you- That- For you: that makes
		sense does it. ((nod))
7	PT:	Yes. ((nod)) **The only thing that**
		**doesn’t make sense is why:(.)**
8		**I’m feeling unable to eat:. [And**
		**restricting what I’m eating.**
9	PR:	[Mm::.
		((nod))
10	PR:	Okay.
11	PT:	And having (.) um (.) ((voice breaks))
		kind of unpleasant
12		thoughts about my body shape? [and,
13	PR:	[Mm:.
		((nod)) Okay.
14	PT:	that.

Sasha describes feeling “unable” to eat and that it “doesn’t make sense” why she is experiencing these thoughts and behaviors. Sasha frames her food restriction as a serious problem, something she cannot control and needs help to address. In Extracts 9B, C, the practitioner indicates that the food restriction is not yet serious, something she may be able to control, and something she already has the resources to address. Extract 9B occurs immediately after Extract 9A.

**Extract 9B:** 

15	PR:	Alright, Okay, **And I assume that**
		**you’re rea:lly (.) try:ing?**
16		eating, ((nod)) as in you’re (.) you
		know trying to give
17		yourself permission (.) to (.) you
		know, enjoy food.Whatever.
18		(.) ‘Cause I guess if you’re quite
		slim and you’re worried
19		about losing more weight. **Now’s not**
		**((shakes head)) the time**
20		**to** start thinking Well I shouldn’t
		have any custard ((smiles))
21		or I [shouldn’t have any-So you’re
		trying t-**Are you trying to**
22	PT:	[((looking down, nods)) °Mm.°
23	PR:	**just have what you fa-fancy when**
		**you-when you could (.) eat**
24		**it.**
25	PT:	I- ((shakes head))
26	PR:	Again it’s e:asier said than [done
		but,
27	PT:	[Whatever
		it is it’s not letting
28		me.
29	PR:	**It’s not what, [Sorry.**
30	PT:	[It’s not letting me.
31	PR:	**Right.**
32		**(2.0)**
33	PR:	Okay.
34	PT:	Like I- (1.0) haven’t eaten anything
		today,
35	PR:	Mm.
36	PT:	And I’ve barely eaten anything since
		Monday, [Just-
37	PR:	[Okay.
38	PT:	Yeah. It’s got out- out of control.
39	PR:	**Mm::. Okay, ((nods, looks away))**
40		**(1.0)**

**Extract 9C:** 

41	PT:	But I feel like no one’s gonna take me
		seriously until I’m
42		underweight. Which- (1.0) I don’t know.
		I’ve=
43	PR:	**=So you’re gonna make yourself**
		**underweight,So people take you**
44		**seriously, Is that’ what you’re=**
45	PT:	=I don’t want that to happen. ((shakes
		head))
46	PR:	No. | We wouldn’t either.
47	PT:	| I don’t want that to be the
		deciding factor in whether I
48		get help for it or not.
49	PR:	Mm:. ((nod))
50	PT:	But I know it’s tricky ‘cause there’s
		so many people °needing
51		help.°
52	PR:	I was gonna say ((nod)) if you think
		there’s a wait for
53		anxiety.
54	PT:	Exactly.=
55	PR:	=and mood problems, it- you know- eh
		for- **for the earlier**
56		**stages of catching and diagnosing**
		**eating disorder it’s- it’s**
57		**wo:rse and longer than that. So have**
		**you got anybody**
58		**supporting you: about eating. Anyone**
		**prompting: you: or**
59		**willing to sit with you:,**

The practitioner immediately asks Sasha to confirm she is “try:ing” to eat and to give herself permission to “enjoy food” (lines 15–17). The question communicates an assumption that Sasha has the choice to try to enjoy food. This does not align with Sasha’s previous descriptions that she is unable to eat (Extracts 9, 10). The practitioner then tells Sasha that “now’s not the time” to think that she should restrict her food (lines 18–21).

**Extract 10:** 

1	PT:	I just always think ‘A:ctually I’ll
		go jump in front of the
2		tra:in.’ [or whatever I’m doing.
**3**	**PR:**	**[Mhm. ((nods, eye contact))**
4	**PR:**	**((continues nodding)) (0.5)**
5	PT:	Yea:h. ((wipes face))
6	**PR:**	**((continues nodding)) (1.0)**
7	PT:	Yeah that’s- that’s the kind of
		thought I have.
8	PR:	**Mhm. It’s a sca:ry thought.**
9	PT:	I kno:w. [It’s ho:rrible.
10	PR:	[((nods))
11	PT:	Or I’ll be like, my anxiety will be
		ba:d. So (.) even when I’m
12		like (.) around the ho:use, [and I
		pick up a knife, [I’m like
13	PR:	[((nods))
		[((nods))
14	PT:	>Okay I can just do this< now,
		[Or like (.) I can just hang
15	PR:	[((nods))
16	PT:	myself now, [I just- It’s just like
		always going on in…
17	PR:	[((nods))

Sasha pushes back on the presupposition that she has the choice to “try” to eat (lines 27–28). She frames the problem as a force outside of herself “Whatever it is….it’s not letting me.” The practitioner does not show agreement or affiliation and responds with minimal acknowledgment (“Right.”) and silence (lines 31–32). Sasha expands on her answer, providing an illustration (lines 34 and 36). She summarizes that her eating has gotten “out of control.” The practitioner minimally accepts (line 39) but does not agree with or validate her experience. The practitioner looks away and there is a long silence.

In Extract 9B, the practitioner subtly communicates a stance that Sasha’s food restriction is not yet serious and is something she may be able to control. Extract 9C occurs immediately after Extract 9B.

After the practitioner’s minimal response (Extract 9B, lines 39–40) Sasha says she feels she will not be taken seriously until she is underweight (Extract 9C, lines 40–41). This also implies that the current practitioner is not taking her problem seriously. The practitioner resists this with an accusation, asking Sasha to confirm that she plans to “make” herself underweight so people will take her seriously (lines 43–44). This again recharacterizes Sasha’s food restriction as within her control and implies that she may try to exploit this intentionally. Sasha again pushes back, stating that she does not want her weight to be the deciding factor in whether she receives care (lines 47–48).

Sasha acknowledges the burden on eating disorder services (lines 50–51) and the practitioner emphasizes the length of the waiting list for eating disorder services (lines 52–53 and 55–57). She describes the wait as “wo:rse and longer” than anxiety disorder services if a person is in “the earlier stages” of an eating disorder. Sasha has not described her eating problems as “earlier stages,” so this further works to minimize and recharacterize her concerns. The practitioner then transitions to ask about friends and family supporting her at mealtimes (lines 57–59). Throughout the rest of the assessment, the practitioner repeatedly encourages Sasha to seek out social support (e.g., “it would be really good to collaborate with somebody in a bit of a buddy way”).

Sasha did not receive a referral for specialist eating disorder services. After attending the ED, Sasha was encouraged by her parents to continue to seek specialist support and began treatment with an eating disorders specialist 3 months later. By then, she had lost a substantial amount of weight. In a 3-month post-visit interview, Sasha reported: “I did get the impression that some people weren’t taking me seriously because I still looked vaguely normal… I’ve lost even more weight since then so kind of firmly within the anorexic range. So I think if – I don’t know – Maybe if I’d been able to access the help sooner then it wouldn’t have got to that stage.”

### Accepting and validating people’s experiences

Below, we present two cases where patients’ experiences were acknowledged, accepted (rather than contested or recharacterized), validated and where practitioners worked to develop a shared understanding with the patient about their experiences.

### Case 4 Emily: accepting and validating the patient’s thoughts of suicide

Emily presented to the ED with suicidal thoughts. In Extract 5, she describes feeling “I might be better off dea:d” but is seeking help because “I don’t want to hurt anyone.” In this section, we present brief extracts from Emily’s video-recorded biopsychosocial assessment and her 1-week post-visit interview.

Emily describes her suicidal thoughts in lines 1–2. The practitioner immediately accepts her description (line 3) and continues to nod as she gives Emily space to continue (lines 4 and 6). Nodding conveys affiliation, i.e., understanding and support of the person’s perspective (44). The practitioner then validates her perspective by acknowledging these thoughts are “sca:ry” (line 8).

Emily does not show signs of disengagement (as in Extract 3) (31) or push back against the practitioner’s response (as in Extract 4). She indicates this is a shared understanding of her experience (“I kno:w”) and aligns with the practitioner’s description (“sca:ry”) by offering a similar upgraded description (“ho:rrible”) (45).

Emily did not describe her suicidal thoughts further when given the opportunity at lines 4/6. However, immediately after the practitioner acknowledges her thoughts as scary, Emily shows a willingness to disclose more sensitive information, describing similar thoughts about ending her life in other ways (lines 11–12, 14, and 16).

In a post-visit interview, Emily described the assessment itself as “really really useful,” particularly “getting off my chest how I was feeling.” Emily reported she “felt quite safe when I went home” because of the conversations she had with this practitioner.

### Case 5 Sam: building on the patient’s characterization of his experience leading up to suicide attempt

It is common in mental healthcare encounters to negotiate about the meaning of and recharacterize a person’s experiences in a more positive way. For example, practitioners can work to reframe patients’ negative thoughts about themselves to facilitate a different understanding (46). Cognitive reframing is a therapeutic tool commonly used to manage negative assumptions and automatic thoughts (47), wherein the practitioner challenges the thought process and introduces alternatives. For example, a practitioner might challenge a patient’s assumption that nothing will help them. This does not involve denying the person’s emotions (e.g., hopeless) or experiences (e.g., of treatment-resistant depression).

In Extract 11, the practitioner introduces a new way of understanding the thoughts Sam experienced before attempting suicide. Sam was brought to the ED after an overdose with suicidal intent. He recently left the army and moved back to his mother’s house. We present brief extracts from Sam’s video-recorded biopsychosocial assessment and his 1-week post-visit interview.

**Extract 11:** 

1	PR:	**I think, from what you’ve said, that**
		**you’ve been struck by**
2		**a NAT.**
3	PT:	What’s a NAT.
4	PR:	**A NAT is a Negative Automatic Thought.**
5	PT:	Mhm,
6	PR:	And what’s happened, is since you’ve
		left the army
		… ((practitioner lists challenges
		patient is facing))
18	PR:	Yeah? It’s hard for you to get a job,
19	PT:	((nods))
20	PR:	You struggle with your mom, ‘cause
		your mom doesn’t
21		understand the situation,
22	PT:	Yeah.
23	PR:	Yeah?
24	PT:	Mhm.
25	PR:	**So what happens is you get this**
		**build-up of negative thoughts**
26		**in your mind.**
27	PT:	Mhm?
28	PR:	Negative th[oughts. Negative thoughts.
29	PT:	[Yeah.
30	PR:	What happens with the build up of the
		negative thoughts?
31	PT:	Yeah.
32	PR:	Yeah? All of a sudden,
33	PT:	Yeah. Yeah.
34	PR:	**what will happen is, “What the heck.**
		**I’m opening up the ah-”**
35	PT:	Paracetamol.
36	PR:	**“medicine cabinet and I’m gonna take**
		**all the pills.”**
37	PT:	Yeah.
38	PR:	**Those negative thoughts become the**
		**norm then don’t they. It’s**
39		**hard to get out of that sort of**
		**mindset.**
40	PT:	Yeah I guess.
41	PR:	**Yeah. What do you think of that?**
42	PT:	You’re right. One hundred percent
		you’re right.

The practitioner proposes that Sam experienced a negative automatic thought (lines 1–3). He lists challenges Sam described earlier in the visit (e.g., unemployment and relationship with mother) (lines 6, 18, and 20–21) and gives Sam opportunities to confirm that the practitioner understood him correctly (lines 19, 22, and 24). He describes a build-up of negative thoughts (lines 25–26, and 28) and frames the pharmaceutical overdose as an understandable outcome (lines 32, 34, and 36). Sam responds with agreement and shared understanding (lines 29, 31, 33, and 35).

The practitioner does not recharacterize, contest or undermine Sam’s experiences. Instead, he gives these experiences a name and introduces a new way of understanding them. He validates how difficult it can be to stop these thoughts (lines 38–39) and asks what Sam thinks of this understanding (line 41). Sam agrees fully, asserting “One hundred percent you’re right.”

In the post-visit interview, Sam described how he felt after the overdose; “I had no one to talk to, I had nothing to do… and then I spoke to him and the team [liaison psychiatry] and they understood… That’s never happened before in my life. No one has actually understood me.” Sam repeatedly emphasized how important this mutual understanding was and described it as the “most helpful” outcome of the meeting. When asked what he would do if he experienced another suicidal crisis, Sam responded; “Talk to someone first. I wouldn’t do it. I’d talk to someone first.”

## Discussion

We identified communication practices used to either undermine, imply/assert alternative characterizations or accept and validate peoples’ accounts of self-harm and suicidality. At times, these practices were also delivered when speaking over the patient. Practices that undermined or implied/asserted alternative characterizations were: not acknowledging or accepting the person’s account; asking questions that implied inconsistency or implausibility (“Didn’t you tell your GP that you were coping okay?”); juxtaposing contrasting information to undermine the account (“You said you were coping okay before, and now you’re saying you feel suicidal.”); asking questions that asserted a different characterization such as implying they were not intending to end their life because they rang a helpline (“So when you called 111 what were you expecting them to do” “So would you say you took the tablets, at the spur of the moment,” “So it was a more of an impulsive thing, at the time?”); and resisting or directly questioning the person’s account (“Really?”).

Multiple practices were used across the assessment that built on each other to imply or assert that: the person was not really suicidal as they did not look or act like they were suicidal; the person’s decision to attend the ED was not justified; that an overdose was impulsive and the person did not really intend to end their life; that self-harming behavior (restricting eating) was not that serious and should be in the person’s control. Together, they were used to evidence inconsistency or implausibility in patients’ descriptions of their experiences.

Importantly, we also identified communication practices that were used to acknowledge, accept and validate suicidality/self-harm and introduce a new way of understanding suicidal thoughts and a suicide attempt that patients found helpful as reported in post-visit interviews with patients. This involved practitioner continuers (such as “Mhm”) which facilitate the patient in fully describing their experience, maintaining eye contact and other non-verbal feedback especially nodding. This also included validation by explicitly stating that the patient’s experiences were difficult and putting forward a candidate understanding (“It’s a scary thought”) rather than remaining silent or asking questions that were designed to recharacterize, subtly undermine or challenge the person’s account of their experiences.

The current findings contribute to an understanding of how peoples’ accounts of self-harm and suicidality are undermined or accepted, a phenomenon which has been reported by patients and leads to negative consequences for them (4, 5). They also contribute to an understanding of the communication practices used when this does *not* happen, i.e., acknowledgment, acceptance, validation and creating meaning and new understandings. Patients report that feeling listened to and understood is vital for effective relationships with health care practitioners (48). However, many patients feel that they are not understood and feel judged for seeking help (3). The current findings show that acknowledging, accepting and validating peoples’ experiences and developing a shared understanding with the person are critical but often overlooked in mental health assessments.

There are a wide range of – and often overlapping – reasons why peoples’ experiences may be undermined or challenged which practitioners report anecdotally. These include: practitioner emotional discomfort with (repeated) exposure to despair and hopelessness; inadequate training and clinical supervision; compassion fatigue and burnout (a risk for people working in the ED); defensive practice which may be heighted after incidents where a practitioner’s assessment and the patient report are not in agreement and the person ends their life; an “epistemic injustice repeat offender” who prefers encounters where they have the upper hand; vicious cycles, arising when patients sense that a practitioner is subtly resisting their account, leading patients to intensify their symptoms to be taken seriously, leading the practitioner to unintentionally respond by becoming even more skeptical. All of these reasons can contribute to patients being treated in a dehumanizing and counter-productive way.

It is important to consider the ED setting and the risk assessment activities underway in these assessments. EDs in the UK are high-pressure environments with 4 h targets for patients to be seen, treated and admitted or discharged. Assessing a person’s mental state and suicide risk involves more than what people say when they are assessed by mental health practitioners on presenting to the ED. A range of factors are considered including, e.g., the person’s social context (social isolation); life events (e.g., bereavement, divorce, domestic violence, and separation from children); family history (mental health problems and family member death by suicide); reports from family/friends/other clinicians about the person’s behavior and mood; and the person’s non-verbal communication. Sometimes, practitioners may feel there are gaps in the person’s story that need to be filled. This makes for a complex judgment and times when practitioners and patients are not in agreement with each other about the degree of suicidality and corresponding risk management. Such cases where there remains unresolved divergence between practitioners and patients are not rare. They highlight the importance of not privileging the patient’s perspective at the expense of the clinician’s or vice versa, as both are unproductive. Communication that is based on collaboration and allows open discussion where there is a lack of shared understanding and disagreement between practitioners and patients is the aspiration not just in meaningful risk assessment but in healthcare communication in general (49). Alongside the pressures in the ED, the number of people seeking help for mental health problems has risen every year while numbers of hospital beds have decreased (50), This increases the pressure to discharge patients even though practitioners are aware of increasingly limited options for treatment (e.g., few in-patient beds, long waiting lists for referrals, high entry thresholds so many people do not meet the criteria for treatment in mental health services). If a person ends their life, practitioners can be called to give evidence in a coroner’s court. Anecdotally, this results in defensive practice, with practitioners feeling helpless and experiencing “moral injury” as they are working in ways that contradict their moral compass (51).

Previous conversation analytic studies of epistemic injustice in mental health have been conducted in social work and substance use settings. Similar to our findings, Lee et al. (52) found two contrasting patterns (i) the worker aligns with the client, actively listening and working to demonstrate understanding and communicating this understanding back to the client, eliciting a deeper client account (ii) the worker assumes a stance of expert and refutes the client’s account of her experience, ending with the client agreeing with the worker’s version. In the current data, practitioners also worked to get patients to align with their alternative characterization. In a substance abuse setting, Auvinen et al. (53) analyzed a group discussion between two rehabilitation clients, a peer support worker and a social adviser. The discussion was based on a motivational interviewing approach which emphasizes the person’s perspective and motivation to change. They found that sharing experiential knowledge, elaborating on personal experiences and developing intersubjective understanding can provide the conceptual resources for people to understand and describe their experience (thereby avoiding hermeneutical injustice).

The practices we focused on were previously identified in police, courtroom, and political settings (20–24). While in police or courtrooms, they are used to assess innocence or guilt, in the pressurized ED setting situated in a pressurized wider mental health services landscape, they can be used to generate alternative characterizations of peoples’ experiences to justify decisions not to refer to specific mental health services. Practitioners are under pressure not to refer patients to overburdened mental health services (54) and are in a position where they must justify denying care in an under-resourced mental healthcare system [see Beale (51)]. Perhaps because of these pressures and lack of access to further care, an epistemic stance that conveys to patients that they have primary rights to know and report on their subjective experiences is even more important. If not, this risks leaving people feeling invalidated, guilty and negatively judged for seeking support and deters future help seeking (55). It also may lead to people being distrustful and unwilling to share what they think and feel with mental healthcare practitioners if they fear being misunderstood based on previous experiences, making it harder to identify optimal support. Some people may avoid seeking help if they have had a difficult interaction with practitioners, and will then miss out on attaining further support at times of future crisis (42). This is consistent with well-established evidence that positive therapeutic relationships predict better treatment engagement and treatment outcomes (56).

Practitioners describe feeling powerless to help patients navigate exclusionary referral criteria (e.g., not meeting threshold with respect to symptom severity for specialist mental health services, and simultaneously too risky for entry level primary care based services) and long waiting lists (3). At the same time, they are held liable for discharging people that are assessed as high risk of self-harm who subsequently die by suicide. Hence, they are under pressure not to report their clinical assessment of need and risk of harm when treatment is not available. As such, undermining and recharacterizing peoples’ experiences may be unconsciously used to justify no further care where services are unavailable or inaccessible, reflecting a wider context of practitioners as gatekeepers, forced to ration mental health services in the UK National Health Service (43).

### Candidacy for mental health services

Interactions with healthcare practitioners have a substantial impact on peoples’ understanding of their own candidacy for mental health services, i.e., their perceptions of whether they have a problem that needs or deserves professional support, and are entitled to seek care (10). By recharacterizing a person’s experiences (e.g., recharacterizing a suicide attempt as “impulsive,” a person’s food restriction as within their control), through their epistemic status and epistemic stance, a practitioner defines the person’s experience in a specific way, e.g., as “impulsive,” “not really suicidal,” “not serious enough to be in the ED” or “in their control.” As Beale (51) has written “We continue to behave as if risk is both predictable and quantifiable, persuading ourselves that certain stock phrases convey a protective coating. ‘Fleeting thoughts of suicide,’ for example, sometimes seen as the precursor to an ‘impulsive’ suicide attempt or act of self-harm. Although it is not without value to record these things in the course of trying to understand someone’s state of mind, it is important to question the attached meaning. In writing ‘no plans or intent’ we make ourselves feel better about the unpredictable nature of suicide, hanging false hope on thoughts that come and go. Rather than admit that someone might end their life but we don’t know when or how, we purport to know it is unlikely to occur.” Based on this characterization, subsequent decisions not to provide further support/refer on to other services communicates that the person does not need further professional support.

Poor communication can leave patients questioning whether adverse mental health experiences were “all in your head” or “not true” (42), as these recharacterizing communication practices can be subtle and difficult for patients to recognize and contest. Hence, the impact on the person may go beyond claiming that the person does not need further professional support; it conveys that the person has a misplaced understanding of their own adverse experiences as “worse than they really are.” There is an inherent power imbalance and the potential for patients to accept practitioners’ claims at face value. This has a knock-on effect on subsequent help-seeking with patients reporting that when they do not feel their experiences were validated or they feel negatively judged for seeking help, they are less likely to seek help in the future even if their mental health has deteriorated further [see Anonymized (42)]. On a population level, this undermines efforts to promote early intervention and improve long-term mental health outcomes.

#### Hermeneutical injustice

Patients described their subjective experiences using concepts such as feeling miserable or being suicidal. Sometimes, the response was to undermine the appropriateness of those concepts, challenging their use with alleged counterevidence, e.g., when the practitioner implied that the patient could not have felt suicidal when he said he had plans for the evening or that he was able to given the impression he was enjoying things. Similar to Lee et al. (52), at other times, the response was to offer alternative expressions to describe the person’s experiences, expressions that the practitioner found more appropriate, e.g., recharacterizing a suicide attempt as impulsive (because the person called an ambulance after an overdose) when the person had not described it in those terms and to persist with the alternative characterization despite the patient’s resistance. This does not reflect a more nuanced understanding of suicidality that can include complex and conflicting thoughts, i.e., wanting to die coexisting with a fear of death. As a result of these challenges and recharacterizations, patients’ feelings and thoughts as they experience them are minimized in further discussion and decision making. In some cases, the person may defer to the practitioner as the expert and stop using the contested concepts, for example, stop using the term “suicidal.” In this way, patients may be subject to hermeneutical injustice as the practitioner does not accept the person’s descriptions or does not negotiate with the person to develop a shared understanding of their experiences.

#### Testimonial injustice, medical records, and barriers to future access to care

Carel and Kidd (57) argue that people with mental and physical illness are more vulnerable to testimonial injustice because they may be considered “cognitively unreliable, emotionally compromised, or existentially unstable in ways that render their testimonies and interpretations suspect.” For example, when a person reports feeling suicidal, their reports can be questioned and challenged more easily if the person has a known mental health issue. While the practitioner-patient interaction is critical in whether people are treated as credible knowers, what is entered in the person’s medical record is also important. For example, one patient’s suicide attempt was recharacterized as “impulsive” although she did not agree with this. While mental health is by its nature negotiated between patients and practitioners, recharacterizations in medical files are likely to go uncontested and potentially shape other healthcare practitioners’ understandings of the patient. Where recharacterized and downplayed versions of patients’ experiences are recorded, other practitioners may not recognize the patient’s risks or may not consider the need for further support. For example, a practitioner might be less likely to consider providing a referral to eating disorder services if previous practitioners did not record the full extent of food restriction in the medical file.

### Strengths and limitations

This is the first study we are aware of to subject the concept of epistemic injustice to empirical analysis using conversation analysis in mental health assessments for people presenting to the ED with self-harm and suicidality. However, we only analyzed five cases as this was an in depth analysis and assessments lasted up to 90 min. We specifically focused initially on cases where peoples’ accounts were not accepted: hence, this is not intended to be representative of the wider dataset. The data were collected in one service and hence may not be representative of other services. Practitioner professional background and training may impact on communication. Given the small sample size, we could not explore this and it would be important to explore in future studies. While we interviewed patients about their assessment, we did not capture the practitioner’s perspective on each assessment. This would have been helpful to understand their perspective on the patient’s experience and their rationale for how they conducted the assessment. It was a challenge to comprehensively analyze practices across a full assessment. Longitudinal conversation analysis is a rapidly developing field (58) and is highly relevant to analyzing epistemic injustice as multiple communication practices build on each other during an assessment and in a person’s mental health interactions over time with different professionals across multiple settings. Triangulating interactional analysis with interviews was informative in highlighting how each assessment was experienced by the specific patient. The longitudinal perspective also shed light on the downstream consequences for patients and carers of having their experiences undermined.

### Future research

Future research should explore to what extent recharacterization could be minimized through further communication training or unconscious bias training, and to what extent a long-term solution may lie in increasing accessibility of mental health services for people that self-harm and experience suicidal ideation. Future research could triangulate multiple data sources, i.e., observation of interactions along with video-stimulated comments and interviews with patients and practitioners to investigate epistemic injustice more closely and the impacts on patients and practitioners over time. Analyzing interactions using conversation analysis may also shed light on empirical approaches to the study of epistemic injustice in other fields such as philosophy.

## Conclusion

Multiple communication practices were used to evidence inconsistency or implausibility in patients’ descriptions of their experiences across the assessment. At times, this included speaking over the patient during their accounts. These practices built on each other to imply or assert that: the person was not really suicidal as they did not look or act like they were suicidal; the person’s decision to attend the ED was not justified; that an overdose was impulsive and the person did not really intend to end their life; that restricting eating (in the context of an eating disorder) was not that serious and should be in the person’s control. Nodding and other non-verbal feedback was central in acceptance of patients’ accounts. These findings have important clinical implications: patients report that when their experiences are not accepted or undermined, this makes them more distressed, less hopeful about the future and discourages future help-seeking when in crisis. Conversely, acknowledging, accepting and validating suicidality/self-harm and introducing a new ways of understanding peoples’ experiences may make people less suicidal and more hopeful, generates shared understanding and encourages future help-seeking.

## Data availability statement

The original contributions presented in this study are included in the article/supplementary material, further inquiries can be directed to the corresponding authors.

## Ethics statement

The studies involving humans were approved by the London Central Research Ethics Committee (17/LO/1234). The studies were conducted in accordance with the local legislation and institutional requirements. The participants provided their written informed consent to participate in this study.

## Author contributions

RM and CB led on conception of the study, with input from all other authors. RM collected the data. CB led the data analysis and all other authors participated in data analysis. CB wrote the first draft of the manuscript with sections written by LB and RM. RM and CB led on manuscript revision with all other authors contributing to manuscript revision. All authors contributed to the article and approved the submitted version.

## Appendix

**APPENDIX TABLE 1:** Transcription conventions.

.hhh	Audible inhalation
hhh	Audible exhalation
:	Extended sound
-	Rising intonation
−	Falling intonation
?	Rising inflection
____	Emphasis (word or part of word underlined)
°°	Talk is quieter than the surrounding talk
<>	Talk is faster than the surrounding talk
UPPERCASE	Talk is louder than the surrounding talk
!	Animated tone
=	Latched utterance, no interval between utterances
[]	Beginning and end of overlapping talk
()	Transcriptionist doubt
(.)	A pause of less than 0.2 s
(0.0)	Silence measured in seconds and tenths of seconds
